# 1,5‐anhydroglucitol predicts the risk and possible mechanisms of Alzheimer’s disease

**DOI:** 10.1002/alz.094722

**Published:** 2025-01-09

**Authors:** Dinghao An, Yun Xu

**Affiliations:** ^1^ Peking Union Medical College, Beijing China; ^2^ Affiliated Drum Tower Hospital of Medical School, Nanjing University, NANJING, Jiangsu China

## Abstract

**Background:**

Alzheimer’s disease (AD) is associated with abnormal glucose tolerance. However, the contributing factors are unknown. We aim to find markers of Alzheimer’s disease in association with abnormal glucose tolerance.

**Method:**

Using genome‐wide association data, we screened out the association between the latest AD dataset and 50,044 datasets to find indicators of glucose tolerance that may be related to AD. Then we implemented a bidirectional MR analysis to evaluate the genetic relation between them and AD. According to the expression levels in brain tissue, we identified the hub genes. Then, we conducted enrichment analysis by combining hub genes and AD drug targets interaction analysis to evaluate the possible pathways. Moreover, we performed a mediation analysis to assess whether blood glucose‐related indicators and diseases are mediators between the two.

**Result:**

After a thorough screening, we found 1,5‐anhydroglucitol, a blood glucose monitoring indicator, could act as a genetically determined risk factor for AD. (ivw: OR = 1.006, 95%CI:1.000‐1.012, p = 0.049; ivw fe: OR = 1.006, 95%CI:1.002‐1.010, p = 0.007). However, we did not find any blood glucose‐related indicators and diseases that could act as a mediating factor. Through genetic annotation of SNP and based on their expression levels in brain tissue and cells, only MGAM and RAB3GAP1 may act as hub target genes, while SI cannot be a target. Hub gene self‐interaction and AD drug target interaction analyses suggest the presence of reported AD‐related genes. Functional and signaling dual pathway analyses suggest the possible mechanism between 1,5‐anhydroglucitol and AD.

**Conclusion:**

We found that 1,5‐anhydroglucitol, a potential marker of glucose tolerance, may be a risk factor for Alzheimer’s disease. We also discovered a potential new target gene for AD: RAB3GAP1 and evaluated possible pathways between 1,5‐anhydroglucitol and AD. Anyway, our findings contribute to the understanding of pathogenesis and yield novel potential therapeutic targets of AD.

